# Integrated miRNA-mRNA Analysis Revealing the Potential Roles of miRNAs in Chordomas

**DOI:** 10.1371/journal.pone.0066676

**Published:** 2013-06-24

**Authors:** Cheng Long, Liang Jiang, Feng Wei, Chuan Ma, Hua Zhou, Shaomin Yang, Xiaoguang Liu, Zhongjun Liu

**Affiliations:** 1 Department of Orthopedics, Peking University Third Hospital, Haidian, Beijing, China; 2 Spine Tumor Center, Peking University Third Hospital, Haidian, Beijing, China; 3 Pathology Department, Peking University Health Science Centre, Haidian, Beijing, China; NIGMS, NIH, United States of America

## Abstract

**Introduction:**

Emerging evidence suggests that microRNAs (miRNAs) are crucially involved in tumorigenesis and that paired expression profiles of miRNAs and mRNAs can be used to identify functional miRNA-target relationships with high precision. However, no studies have applied integrated analysis to miRNA and mRNA profiles in chordomas. The purpose of this study was to provide insights into the pathogenesis of chordomas by using this integrated analysis method.

**Methods:**

Differentially expressed miRNAs and mRNAs of chordomas (n = 3) and notochord tissues (n = 3) were analyzed by using microarrays with hierarchical clustering analysis. Subsequently, the target genes of the differentially expressed miRNAs were predicted and overlapped with the differentially expressed mRNAs. Then, GO and pathway analyses were performed for the intersecting genes.

**Results:**

The microarray analysis indicated that 33 miRNAs and 2,791 mRNAs were significantly dysregulated between the two groups. Among the 2,791 mRNAs, 911 overlapped with putative miRNA target genes. A pathway analysis showed that the MAPK pathway was consistently enriched in the chordoma tissue and that miR-149-3p, miR-663a, miR-1908, miR-2861 and miR-3185 likely play important roles in the regulation of MAPK pathways. Furthermore, the Notch signaling pathway and the loss of the calcification or ossification capacity of the notochord may also be involved in chordoma pathogenesis.

**Conclusion:**

This study provides an integrated dataset of the miRNA and mRNA profiles in chordomas, and the results demonstrate that not only the MAPK pathway and its related miRNAs but also the Notch pathway may be involved in chordoma development. The occurrence of chordoma may be associated with dysfunctional calcification or ossification of the notochord.

## Introduction

Chordomas are rare, slow-growing, primary malignant neoplasms of the axial skeleton and arise from the remnant notochord [1]–[3], and surgery remains the best standard treatment [3], [4]. However, these tumors are difficult to be eradicated because they are often adjacent to vital structures. Accordingly, the prognosis of patients with chordomas is often poor; many patients develop fatal local recurrence [5], and the overall median survival is 6.29 years [1]. Therapeutic advances are therefore urgently required for improving the outcome.

MicroRNAs (miRNAs) are a class of short (18–25 nucleotides) noncoding RNAs that suppress translation, increase mRNA deadenylation and degradation, and/or sequester the mRNA of target genes [6]. It is estimated that up to 30% of human genes [7] and virtually all cellular processes are regulated by miRNAs [8]. Abnormal expression of several miRNAs has previously been shown to be associated with multiple cancer types [9], including chordomas [10]. However, no studies have applied integrated analysis techniques, which can be used to identify functional miRNA-target relationships with high precision to miRNA and mRNA profiles for chordomas.

In this study, we applied an integrative molecular and bioinformatic approach by simultaneously profiling both miRNA and mRNA for chordomas and notochord tissues to investigate the mechanisms responsible for the progression and pathogenesis of chordomas. The microarray data were validated by quantitative real-time reverse transcription polymerase chain reaction (qRT-PCR). The understanding of the molecular differences between chordoma and the notochord may shed light on the molecular pathogenesis of chordoma and offer new possibilities for systemic treatment.

## Materials and Methods

### 2.1 Ethics Statement

Our study design received approval from the institutional review board of Peking University Third Hospital (Beijing, China) (No. IRB00006761–2012039). Written informed consent was obtained from the patients.

### 2.2 Tissue Samples

**Figure 1:**
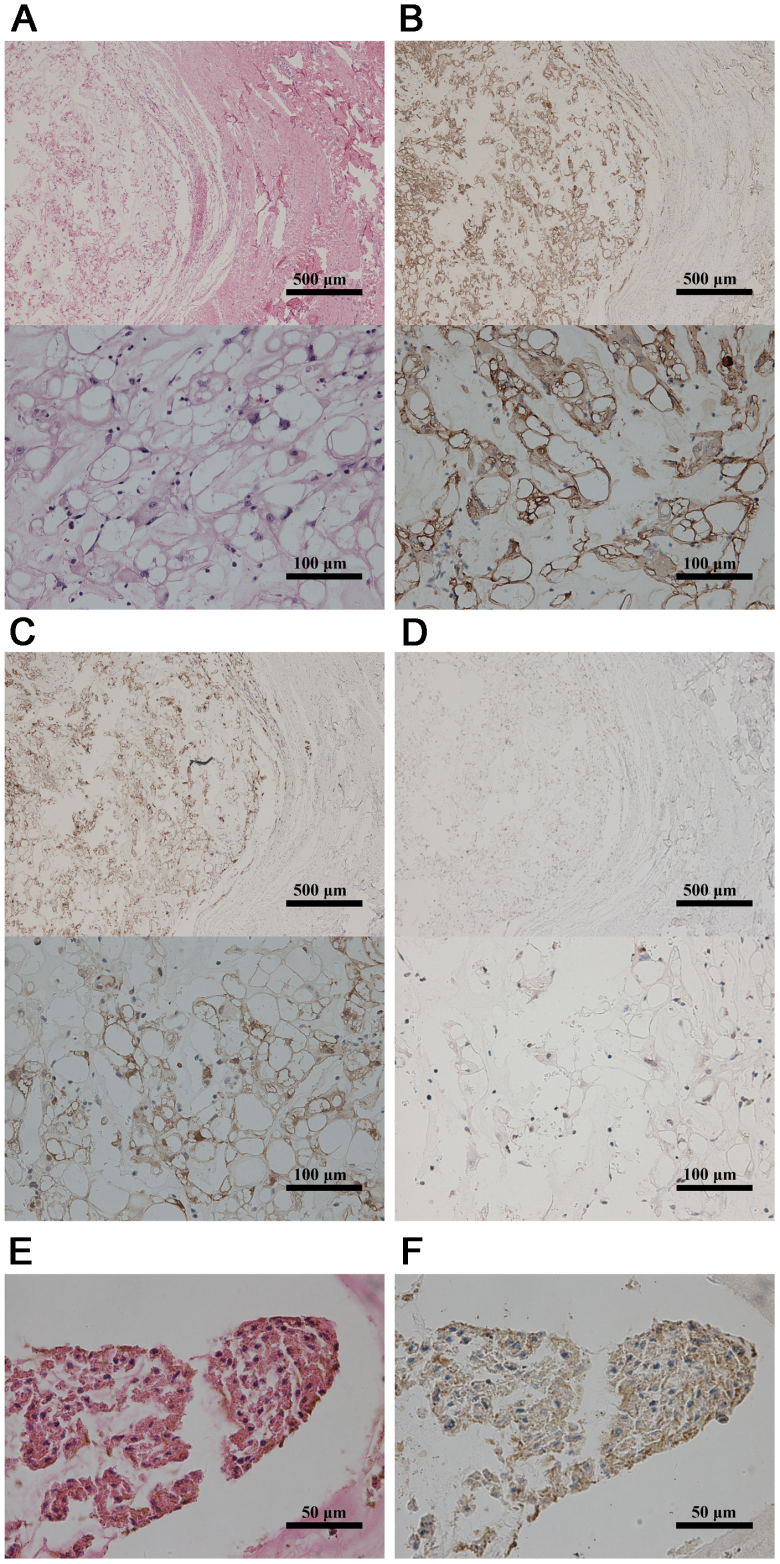
Identification of chordoma and notochord tissues. H&E stained section of a chordoma (A) showing moderately atypical physaliphorous (intracellular, bubble-like vacuoles) cells set within a myxoid matrix. The tumor cells demonstrated positive immunostaining for cytokeratin (B), S100 protein (C), and brachyury (D). H&E stained section of a notochord tissue (E) showed a clear boundary between the notochord and the surrounding tissue. The notochord cells demonstrated positive immunostaining for brachyury (F).

Three pairs of paraformaldehyde-fixed, paraffin-embedded (PFPE) tissue samples were divided into 2 groups (Table S1). One group contained three primary classic chordoma tissues (obtained from men with a mean age of 43.3 years; chordoma group), and the other group contained three notochord samples obtained from the intervertebral discs of aborted fetuses with a gestational age of 24–27 weeks (notochord group). Paraffin sections from the fixed chordoma tissues were cut at 5 µm and stained with hematoxylin and eosin (H&E) as well as antibodies against cytokeratin, S100 and brachyury proteins [11]–[14] (Figure 1). The sections of fetal notochord were also stained with H&E and received immunohistochemical study with brachyury proteins (Figure 1). These samples were confirmed by two experienced pathologists.

### 2.3 Laser Capture Microdissection

All tissues were separated using laser capture microdissection (LCM). Briefly, a series of 10-µm-thick sections was cut from paraffin-embedded tissues and the sections were affixed to cross-linked polyethylene foils that were attached to glass slides (Leica, Wetzlar, Germany). The slides were then further processed using H&E staining. A Leica AS LMD microdissection system (Leica) was used to capture samples from the chordomas and notochord tissues. Target tissue samples identified by H&E staining were outlined by free-hand tracing, cut from the slide by the laser and collected into the cap of a 0.2 ml PCR tube immediately. All the samples were stored at −80°C until they were processed for RNA extraction in 1 batch.

### 2.4 RNA Extraction and Microarray Experiments

Total RNA was isolated from PFPE samples by using RecoverAll™ Total Nucleic Acid Isolation Kit (Life Technologies, Carlsbad, CA, USA) for mRNA and miRNA microarray analysis, according to the manufacturer’s instructions. Total RNA quality and integrity were confirmed by denaturing gel electrophoresis. Total RNA was purified using RNeasy Mini Kit (Qiagen, Hilden, Germany) and amplified using a sensation kit (Genisphere, Hatfield, PA, USA). We ensured that the purification method retained low-molecular-weight (LMW) RNA. MiRNA expression profiling was performed using Affymetrix Gene Chip miRNA 2.0 arrays (Santa Clara, CA, USA) containing 1,105 human mature miRNAs in miRBase 15 (http://microrna.sanger.ac.uk). Messenger RNA expression profiling was performed using Affymetrix GeneChip Human Gene 1.0 ST Array (Santa Clara), which contains 764,885 probe sets from 28,132 genes (Ensembl) or from 19,734 putative full-length transcripts (GenBank and Ref Seq).

Briefly, for miRNA expression profiling, the RNA was labeled with FlashTag Biotin HSR (Genisphere) and then hybridized to Affymetrix miRNA array. After hybridization, staining and washing were performed according to the user guide. For mRNA expression profiling, the RNA was reverse transcribed to double-stranded cDNA, fragmented and labeled with Biotin labeling kit (Genisphere), and then hybridized to Affymetrix gene 1.0 array as recommended. Standard Affymetrix array cassette staining, washing, and scanning were then performed. The last step was to scan the signals and analyze the data. Affymetrix® Expression Console Software (version 1.2.1) was used for microarray analysis. Raw data (CEL files) were normalized at the transcript level by using a robust multi-average method (RMA workflow). MiRNA and mRNA expression data are available from the NCBI Gene Expression Omnibus (GEO), accession number GSE37372.

### 2.5 Data Analysis

Normalized data from each array were analyzed using two-class differentiation, which is applicable to analyses of small samples. We applied the random variance model (RVM) t-test to filter differentially expressed miRNAs and mRNAs for the 2 groups [15]. Fold change and the false discovery rate (FDR)-adjusted *P* values (*P*<0.05) were used to screen miRNAs and mRNAs with significantly different expression.

Hierarchical clustering of miRNAs and mRNAs with significantly different expression was performed using the Cluster 3.0 software and visualized with Treeview v1.60.

### 2.6 Integrated Analysis of miRNA Targets

The differentially expressed miRNAs were then selected for target prediction by using TargetScan database version 6.0 (http://www.targetscan.org/). To improve the accuracy of target prediction, we further combined the analysis of differentially expressed mRNA with target prediction of the differentially expressed miRNAs. The intersecting gene set was subject to bioinformatic analysis.

### 2.7 Bioinformatic Analysis

We applied the Gene Ontology (GO) classification of genes to determine the functions of the intersecting genes and uncover the miRNA-gene regulatory network on the basis of biological process and molecular function. In detail, the two-sided Fisher’s exact test and χ^2^ test were used to classify the GO category, and the FDR was calculated to correct the *P* value. We chose only GOs that had *P*<0.01.

To identify the pathways of intersecting genes, Kyoto Encyclopedia of Genes and Genomes (KEGG, http://www.genome.ad.jp/kegg/) enrichment analysis was performed. This analysis provides a better understanding of gene expression information as a complete network. The Fisher’s exact test, χ^2^ test, and the threshold of significance were defined by the *P* value and FDR. The screening criterion was *P*<0.05.

### 2.8 qRT-PCR of miRNAs

The microarray data were validated by qRT-PCR. Specific bulge-loop™ miRNA qRT-PCR primer sets (1 reverse transcription primer and a pair of quantitative PCR primers for each set) were designed by RiboBio (Guangzhou, China). RNU6B (Guangzhou RiboBio Co., Ltd) was used as the internal control. RNU6B is a small nuclear RNA that is frequently used as reference RNA for miRNA quantification. RT-PCR reactions were conducted according to the manufacturer’s recommendation. In brief, reverse transcriptase reactions contained purified total RNA, RT primers for each miRNA and U6 small nuclear RNA, RT buffer, dNTPs, RNase inhibitor, DTT, and M-MLV reverse transcriptase. qRT-PCR was performed using SYBR-Green PCR Master Mix (Takara) and real-time cyclers (Strata Gene MX3000P qPCR system). The PCR cycling conditions were as follows: 95°C for 10 min, followed by 40 cycles of denaturation at 95°C for 15 s and a combined annealing/extension step at 60°C for 60 s. The reaction was conducted using the real-time thermal cycler Mx3005p from Agilent Technologies (Agilent Technologies, Waldbronn, Germany). All parameters were measured in triplicate.

## Results

### 3.1 miRNA Array Analysis and Target Prediction

**Figure 2:**
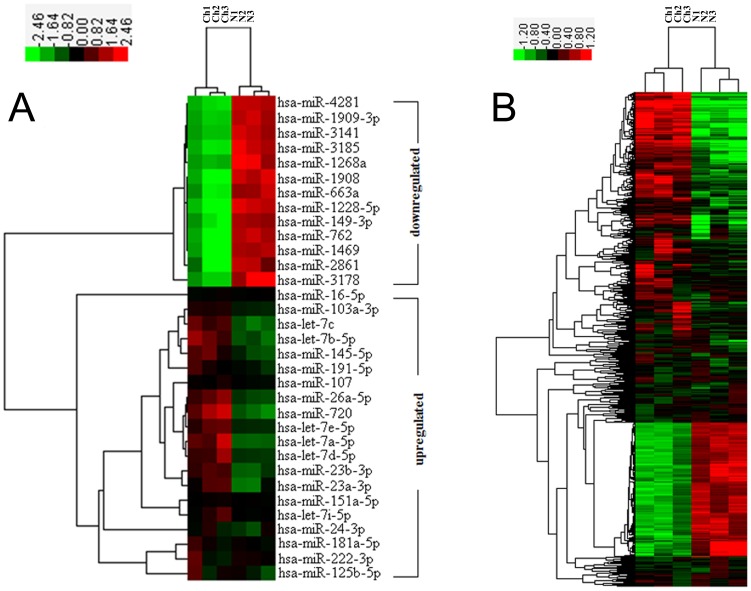
Hierarchical clustering of differentially expressed miRNAs and mRNAs in chordoma tissues (Ch1, Ch2, Ch3) and notochord tissues (N1, N2, N3). (A) The 33 miRNAs listed above were differentially expressed (*P*<0.05) between the chordoma tissues and notochord tissues. (B) In total, 2,791 mRNAs differed between the two sample groups. The color scale shown on the top illustrates the relative expression level of the indicated miRNA across all samples: red denotes high expression levels, whereas green denotes low expression levels.

In our study, 33 (20 upregulated and 13 downregulated) of the 1,105 analyzed miRNAs were significantly dysregulated in the chordoma group relative to the fetal notochord group (Figure 2A, Table S2). To further determine the biological functions of these miRNAs, TargetScan was used to predict the target genes of the 33 miRNAs, which resulted in the identification of 6,045 putative target genes (Table S3).

### 3.2 mRNA Array Analysis and Integrative Identification of miRNA Targets

The mRNA array showed that 2,791 mRNAs were differentially expressed, including 577 mRNAs that were downregulated and 2,214 mRNAs that were upregulated in chordomas relative to the fetal notochords (Figure 2B, Table S4). Among these genes, 911 overlapped with putative target genes of differentially expressed miRNAs, including 87 downregulated mRNAs and 824 upregulated mRNAs (Table S5). These 911 intersecting genes were subjected to bioinformatics analysis.

### 3.3 GO Analysis

**Figure 3:**
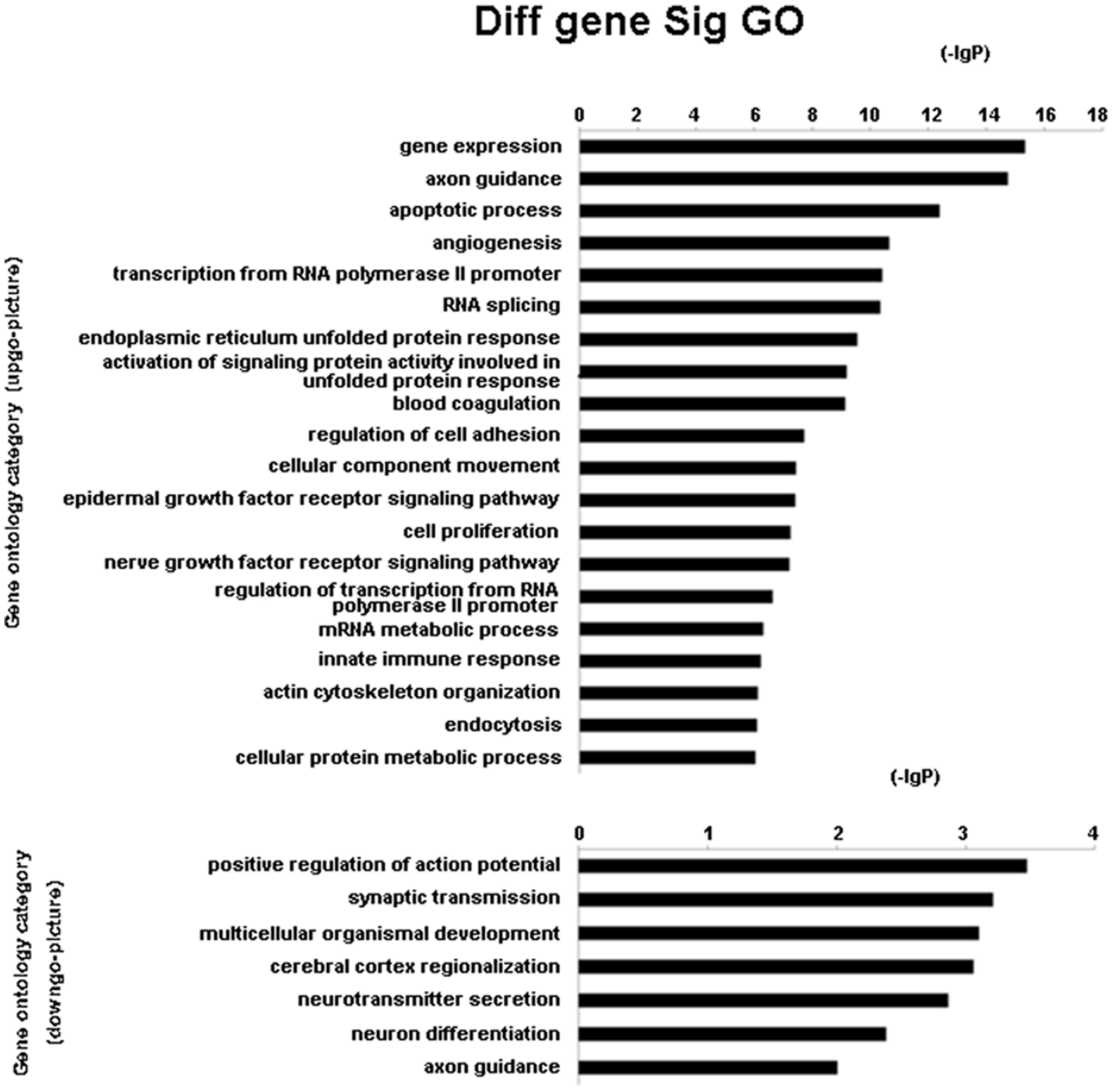
miRNA targeted significant GOs. The upper chart shows the GOs targeted by downregulated miRNA, and the lower chart shows the GOs targeted by overexpressed miRNA. The vertical axis is the GO category and the horizontal axis is the -lg p value of the GO category.

GO enrichment analyses indicated that 7 GOs were significantly regulated by the downregulated genes, whereas 184 GOs were significantly regulated by the upregulated genes. The main GO categories targeted by the upregulated genes included gene expression, axon guidance, and apoptotic processes (Figure 3). In contrast, significant GOs corresponding to the downregulated genes included positive regulation of the action potential, multicellular organismal development, and cerebral cortex regionalization (Figure 3).

### 3.4 Pathway Analysis

**Figure 4:**
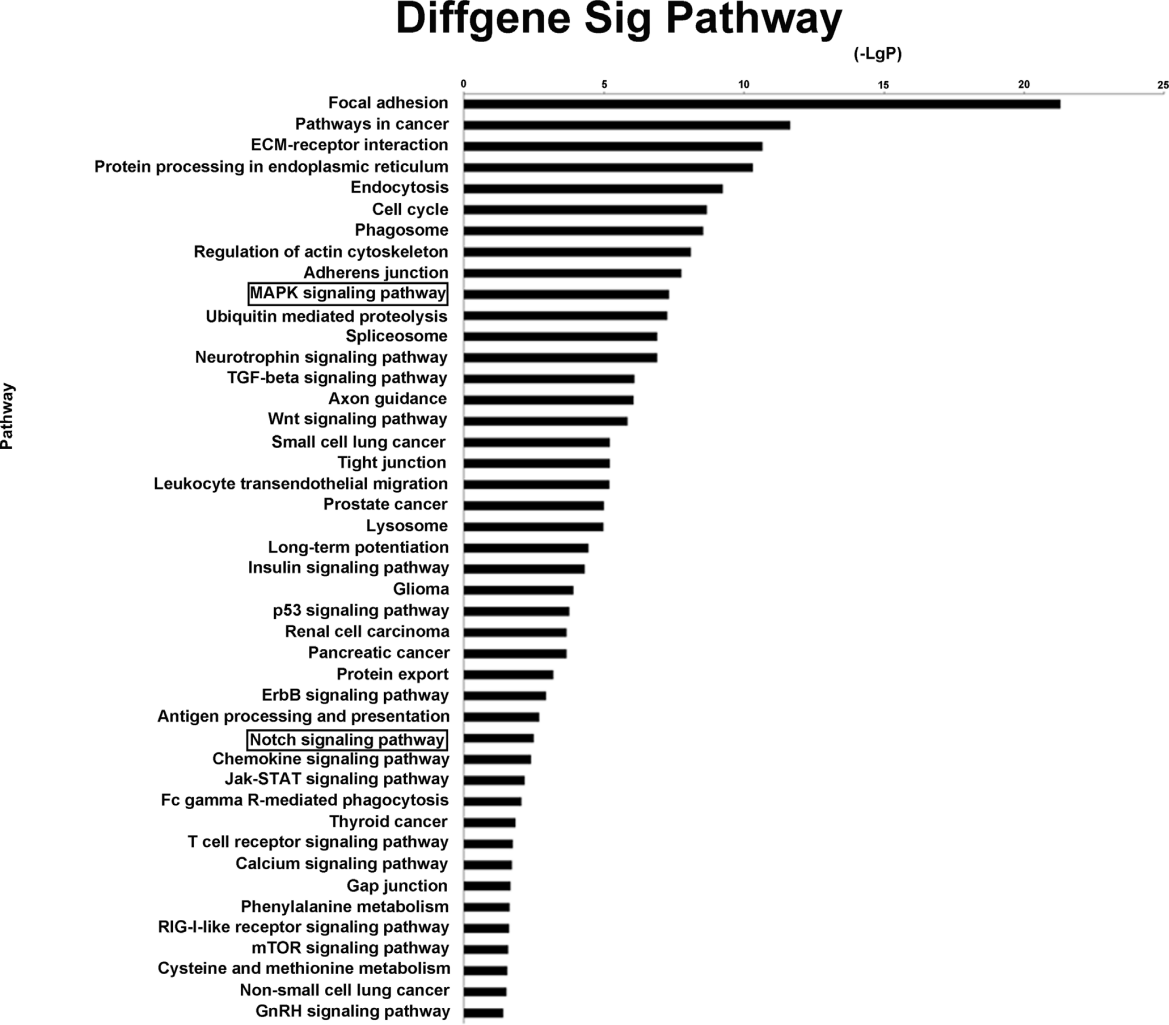
Pathway analysis based on miRNA-targeted genes. Significant pathways targeted by downregulated miRNA are shown. The vertical axis is the pathway category, and the horizontal axis is the enrichment of pathways.

Pathway analyses showed that 44 different pathways corresponded to the significantly upregulated intersecting genes. Overall, a genetic cluster summarizing the functions of focal adhesion, pathways in cancer, and ECM-receptor interactions was found to have the highest relationship with the chordoma group (Figure 4, Table S6).

By considering the genetic pathways listed in KEGG as being involved in cancer development, we identified several significantly related pathways, including MAPK signaling, neurotrophin signaling, TGF-beta signaling, Wnt signaling, insulin signaling, p53 signaling, ErbB signaling, Notch signaling, chemokine signaling, Jak-STAT signaling, T cell receptor signaling, calcium signaling, RIG-I-like receptor signaling, mTOR signaling, and GnRH signaling (Figure 4, Table S6).

In addition to these classical pathways, several clusters of genes associated with the following major cancer entities were overrepresented, which suggests a common oncogenic basis: small cell lung cancer, prostate cancer, glioma, renal cell carcinoma, pancreatic cancer, thyroid cancer, and non-small cell lung cancer (Figure 4, Table S6).

Notably, the Notch signaling pathway was dysregulated in chordoma; aberrant Notch signaling is associated with tumorigenesis in many types of tumors [16], [17]. Six genes (*NOTCH2, NCOR2, CREBBP, JAG1, KAT2A* and *NCSTN*) related to the Notch signaling pathway were upregulated in chordoma tissues.

### 3.5 Validation of miRNA Array Data

To validate the microarray data, 7 miRNAs were selected and subjected to qRT-PCR validation. Our pathway analysis showed that the most highly overrepresented genetic pathway involved in chordoma development was the MAPK signaling pathway, which had the lowest *P* value (*P = *4.79E-8). Given that constitutive activation of the MAPK signaling pathway plays a pivotal role in various human neoplasms [18] and that the MAPK signaling pathway has been association with chordomas [19], our findings prompted us to perform analyses to identify miRNAs with the potential to target the MAPK pathway.

**Figure 5:**
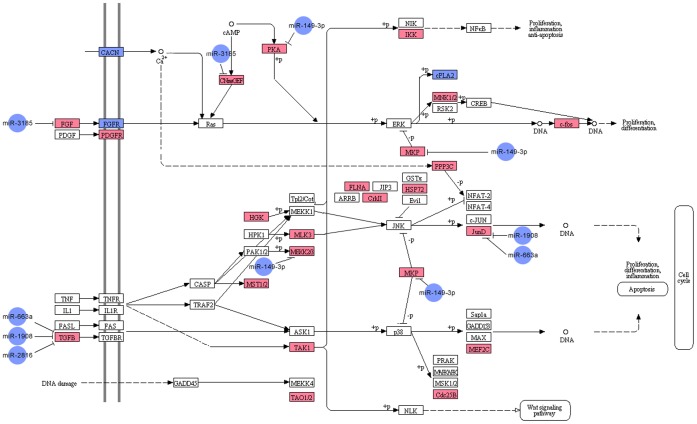
miRNA-gene network of the MAPK signaling pathway in chordomas. Blue box nodes represent downregulated mRNAs, pink box nodes represent upregulated mRNAs, and blue cycle nodes represent downregulated miRNAs.

In the set of significantly dysregulated miRNAs, 5 downregulated miRNAs (miR-149-3p, miR-663a, miR-1908, miR-2861, and miR-3185) were predicted to target genes encoding 7 upregulated MAPK signaling pathway-related mRNAs (*FGF2, JUND, DUSP4, MAP3K3, TGFB1, PRKACA* and *RAPGEF2*) (Figure 5). The 5 differentially expressed miRNAs were selected on the basis of their involvement in the MAPK pathway and were subjected to qRT-PCR validation.

Additionally, because chordoma is a primary bone tumor, miR-762 and miR-1228 were also included for validation because they are involved in calcification [20] or osteoblast differentiation [21], [22].

**Figure 6:**
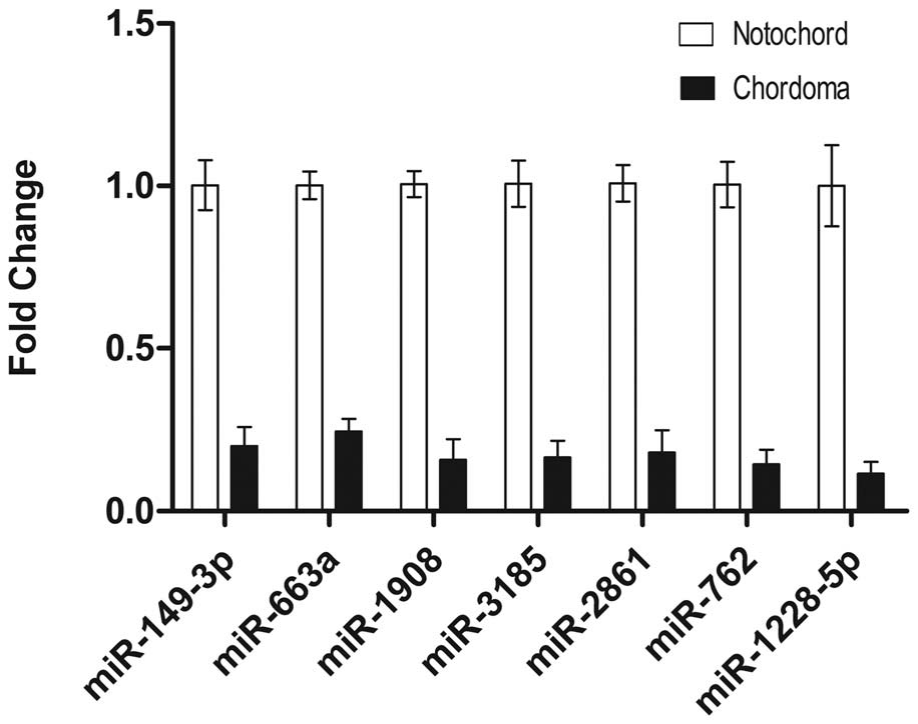
Quantitative analysis of miRNA expression in chordomas. Differentially expressed miRNAs (miR-149-3p, miR-663a, miR-1908, miR-3185, miR-2861, miR-762, and miR-1228-5p) in chordomas (n = 13) relative to fetal notochords (n = 3).

All the 7 miRNAs were present in 13 chordoma samples (including 3 used for microarray analysis) and 3 notochord samples (used for microarray analysis). Differential expression was confirmed for all the miRNAs analyzed, as shown in Figure 6. These 7 miRNAs may therefore play a role in the malignant progression of chordomas.

## Discussion

### 4.1 Integrated miRNA-mRNA Analysis of Chordomas

Cancer is a complex genetic disease that involves structural and regulatory abnormalities in both coding and non-coding genes, and abnormal expression of miRNA appears to be representative of aberrant gene expression in cancer cells [23]. Several miRNAs have been found to be involved in the initiation and progression of several types of human cancers [23]. Duan et al. [10] first established a direct connection between a cell signaling pathway implicated in the molecular pathogenesis of chordoma and the miRNA machinery; they profiled 21 miRNAs that were differentially expressed in chordoma tissues and chordoma cell lines when compared with normal muscle tissues and found that miR-1 and miR-206 were particularly downregulated in chordomas. Overexpression of miR-1 was found to suppress Met expression and inhibit the growth of chordoma cells. Therefore, miRNA-1 was suggested to have a functional effect on the pathogenesis of chordoma. Recently, it has been suggested that paired expression profiles of miRNAs and mRNAs can be used to identify functional miRNA-target relationships with high precision [24]. To our knowledge, the network of miRNA-mRNA interactions in chordomas has not been described. In this study, we have introduced integrated analysis of miRNA and mRNA expression profiles in classical primary chordoma tissues. Our miRNA microarray results revealed a set of miRNAs that are differentially expressed in chordoma tissue when compared with fetal notochord tissue. Our mRNA microarray results showed that *ENO1*, *PKM2,* and *Gp96* were upregulated in chordoma tissue relative to notochord tissue. This result is consistent with our previous findings [25]. However, these genes were not identified as target of the dysregulated miRNAs. These results support our previous findings that ENO1, PKM2, and Gp96 may play roles in chordomas, but also imply that these genes are not directly regulated by any of the 33 differentially expressed miRNAs.

### 4.2 Selection of Fetal Notochord as the Control

Differential miRNA expression profiling analysis of human tumor and normal tissues allows the identification of miRNAs that may provide key information regarding carcinogenesis [10]. Chordomas are the most frequent primary tumors of the spine and account for 1–4% of all primary bone tumors [1], [2]. Chordomas were originally thought to develop from cartilage [1]; however, chordomas localize at the same sites as notochordal remnants [3], and immunohistochemical [26] and ultrastructural [27] similarities between chordomas and notochordal tissue suggest that chordomas arise from remnants of the embryonic notochord [28]. Recently, brachyury, which is crucial in notochord development, was also observed in tumor cells of primary chordomas [14], which provide compelling evidence to support the hypothesis that chordomas originate from the remnant notochord.

In comparative studies of chordomas, it is difficult to find corresponding normal tissues to use as a control. During embryogenesis, most notochordal cells die and are replaced by bone in the vertebral bodies and by the nucleus pulposus in the intervertebral discs [29]. In humans, notochordal cells are believed to disappear by the age of 10 years [30]. Additionally, the nucleus pulposus expresses neither cytokeratins nor brachyury [14], whereas both are coexpressed in the embryonic notochord [14]. Therefore, whether the nucleus pulposus is formed directly by notochordal cells is unclear [29]. In a previous study [10], normal muscle tissues were used as a control. In our study, we choose fetal notochord as a control for chordomas. The previous study identified 4 differentially expressed miRNAs that overlapped with the 33 differentially expressed miRNAs in the present study, including two miRNAs (miR-1228 and miR-1268) with the same downregulation trend that we observed and two miRNAs (miR-23a and miR-26a) with dysregulation trends opposite to those that we observed. We believe that the differences between our results and those of the previous study mainly arise from differences in the control and small sample size. Our microarray results identified a set of miRNAs that were differentially expressed in chordoma tissue relative to fetal notochord tissue. The differentially expressed miRNAs were clearly different between the chordoma and notochord tissue and therefore may, together with their target genes, be involved in the pathogenesis of chordomas. On the other hand, only 3% (33/1,105) of all analyzed miRNAs showed a difference in the expression level, which suggests a close relationship between these 2 tissues. However, it should be emphasized that the differences that we found may be influenced by the different developmental stages for the chordoma and fetal notochord tissues. Additionally, because of the limited number of samples (only 6 tissue samples) in this study, the results may have been influenced by individual differences as well.

### 4.3 Bioinformatics Analysis

GO provides a structured ontology of defined terms representing putative functional properties of specific gene products. KEGG enrichment analyses enable a better understanding of gene expression information as part of a complete network. To gain insights into the functional targets of the 33 differentially expressed miRNAs, GO and KEGG pathway annotation were applied to the putative target gene pool.

The GO analysis showed that the apoptotic process was the critical GO term. Evading apoptosis is an essential alteration for the malignant growth of cells [31], and heightened resistance to apoptosis is implicated in the processes of invasion and metastasis [32]. Therefore, this result was in line with the basic tumor characteristics. KEGG pathway annotation indicated that the MAPK signaling pathway was the most highly overrepresented genetic pathway. The MAPK signaling pathway governs many eukaryotic cellular processes such as proliferation, differentiation, and survival [33], and constitutive activation of the MAPK signaling pathway is a major event in various human neoplasms [18].

Notably, our results showed that the Notch signaling pathway was altered in the chordoma group, which implies that this pathway may also be involved in the pathogenesis of chordomas. There is mounting evidence that this pathway is activated in leukemia and solid tumors and can induce tumor formation [34], [35], and Notch targeting approaches are considered to be a novel molecular therapy for cancer [16]. However, activation of the Notch signaling pathway in chordoma has not been previously reported and requires further study.

### 4.4 MAPK Pathway and Related miRNAs in Chordomas

Recently, several studies demonstrated a critical role of the MAPK signaling pathways in chordomas [19]. Our pathway analyses also showed that the MAPK signaling pathway was the most highly overrepresented genetic pathway. The five significantly downregulated miRNAs (miR-149-3p, miR-663a, miR-2861, miR-1908, and miR-3185) were predicted to target MAPK signaling pathway-related genes (*FGF2, JUND, DUSP4, MAP3K3, TGFB1, PRKACA* and *RAPGEF2*) through an inverse relationship. Four of the five down regulated miRNAs have previously been suggested to be involved in other human cancers. Downregulation of miR-149 has been identified in multiple cancer types, such as colorectal cancer [36], astrocytoma [37], and gastric cancer [38]. Overexpression of miR-149 can inhibit tumor cell proliferation and cell cycle progression by targeting ZBTB2 in gastric cancer cell lines [38], and it can inhibit tumor cell migration [37]. Expression of miR-149 has been associated with tumor invasion depth [36]. Dysregulation of miR-663 has been reported in multiple cancer types [39], [40]. Introduction of miR-663 into the human gastric cancer cell lines BGC823 and SNU5 induced morphological changes and suppressed proliferation of these cells in vivo and in vitro [40]. In addition, miR-663 alters the DNA content and induces phenotypes of mitotic catastrophe in tumor cells [40]. Therefore, miR-149 and miR-663 have been regarded as tumor suppressors [36], [40]. MiR-1908 has been identified as having a close relationship with metastatic invasion, angiogenesis, and colonization of melanomas [41]. Decreased expression of miR-2861 has also been found in basal cell carcinomas [42]. In the current study, we also found reduced expression of miR-149-3p, miR-663a, miR-1908 and miR-2861 in chordomas.

Our results, and the association of miR-149-3p, miR-663a, miR-1908 and miR-2861 with tumorigenesis reported previously in the literature, led us to propose that the 4 miRNAs may play an important role in the malignant progression of chordoma. However, the biological functions of miR-3185 have not been reported earlier and need further study.

### 4.5 The Role of Calcification/Osteoblast Differentiation in Chordomas

Chordoma is a primary bone tumor and originates from remnants of the embryonic notochord, which normally becomes ossified in regions of forming vertebrae and contributes to the center of the intervertebral discs [29], [43], [44]. In the present study, we observed that three miRNAs (miR-762, miR-1228, and miR-2861) that have been previously reported to be associated with calcification or osteoblast differentiation were significantly dysregulated.

MiR-1228 has been found to be upregulated in many malignancies [45], [46] and is involved in the inhibition of cellular apoptosis by repressing MOAP1 expression [47]; however, it was downregulated in our study and in previous miRNA study on chordomas [10]. One possible explanation of this discrepancy is that the same miRNA could have different targets and the same mRNA could be targeted by different miRNAs in different cell types [23]. Thus, the same miRNA can participate in distinct pathways and have different effects on cell survival, growth and proliferation, that are dependent on cell type and the gene expression pattern.

MiR-1228 is also involved in osteoblast differentiation. Evidence suggests that miR-1228 is involved in 1,25-dihydroxyvitamin D (1,25D)-mediated regulatory effects in bones. Inactivation of miR-1228 alone was sufficient to abrogate 1,25D-mediated downregulation of BMP2K protein expression [48]. The role of miR-1228 in chordomas has not been elucidated thus far.

MiR-2861 is another miRNA that has been shown to play an important physiological role in osteoblast differentiation [21], [22]. Overexpression of miR-2861 enhances BMP-induced osteoblastogenesis, and silencing of miR-2861 inhibits bone formation [21].

Altered expression of miR-762 is associated with calcification [20]. A previous study showed that overexpression of miR-762 decreased the protein levels of NCX1, PMCA1, and NCKX4, which are involved in calcium transport [20]. Inhibition of miR-762 restores the protein level of calcium pumps and reduces the degree of Pi- and Ca-induced calcification [20].

Therefore, the three downregulated miRNAs in the chordoma group in our study may have an effect on the calcification or osteoblast differentiation of notochord tissues. Loss of the capability of calcification or osteoblast differentiation may result in the formation of undifferentiated notochordal remnants and play a role in the occurrence of chordoma.

In conclusion, to our knowledge, this investigation is the first to integratively analyze miRNAs that are differentially expressed between chordomas and notochord tissue. Based on preliminary microarray data, we defined a set of miRNA candidates that are dysregulated in chordomas; however, these findings also imply a close relationship between chordomas and notochord tissue in another way. Our results also demonstrate that not only the MAPK signaling pathway and its inversely related miRNAs (miR-149-3p, miR-663a, miR-1908, miR-2861, and miR-3185) but also the Notch signaling pathway may play a role in chordoma development. In addition, the occurrence of chordomas may be associated with the dysfunction of notochord ossification. However, it should be emphasized that the miRNAs and their target genes integratively analyzed in this study were only bioinformatically predicted and should be considered for further validation and functional examination.

## Supporting Information

Table S1 **Details of the primary classic chordoma tissues and control specimens included in this study.**
(DOCX)

Table S2 **miRNAs that were significantly dysregulated in chordomas compared with fetal notochords.**
(DOCX)

Table S3 **Putative target genes of the 33 miRNAs predicted by TargetScan.**
(XLS)

Table S4 **mRNAs that were differently expressed in chordomas relative to fetal notochords.**
(XLS)

Table S5 **Intersecting genes targeted by dysregulated miRNA and encoding dysregulated mRNA.**
(XLS)

Table S6 **Pathways corresponding to the significantly upregulated intersecting genes.**
(XLS)
