# Supplementary material for: Integrated miRNA-mRNA Analysis Revealing the Potential Roles of miRNAs in Chordomas
Source: PLoS One. 2013 Jun 24;8(6):e66676. doi: 10.1371/journal.pone.0066676 (PMC3691184; doi:10.1371/journal.pone.0066676)
Supplement: Table S1 — Details of the primary classic chordoma tissues and control specimens included in this study. (DOCX) [file pone.0066676.s001.docx]

**Table S1.** Details of the primary classic chordoma tissues and control specimens included in this study .

| **Sample ID** | **Histology** | **Sex** | **Age/ gestation age** | **Locale** | **RNA concentration (ng/ul)** | **A260/280 ratio** |
| --- | --- | --- | --- | --- | --- | --- |
| Ch1 | classic chordoma | M | 50y | C2-3 | 276.80 | 1.86 |
| Ch 2 | classic chordoma | M | 41y | C2 | 572.20 | 1.96 |
| Ch 3 | classic chordoma | M | 38y | C3 | 258.60 | 2.01 |
| N1 | notochord | M | 27w | C5-6 IVD | 183.30 | 1.99 |
| N2 | notochord | M | 25w | C5-6 IVD | 144.80 | 2.07 |
| N3 | notochord | M | 24w | C5-6 IVD | 157.20 | 2.04 |

M, male; y, year; w, week; C, cervical; IVD, intervertebral disc; Ch, classic chordoma; N, notochord.
