# Supplementary material for: Integrated miRNA-mRNA Analysis Revealing the Potential Roles of miRNAs in Chordomas
Source: PLoS One. 2013 Jun 24;8(6):e66676. doi: 10.1371/journal.pone.0066676 (PMC3691184; doi:10.1371/journal.pone.0066676)
Supplement: Table S2 — miRNAs that were significantly dysregulated in chordomas compared with fetal notochords. (DOCX) [file pone.0066676.s002.docx]

**Table S1.** 33 miRNAs that sufficient to perfectly separate the chordoma samples from the fetal notochord samples.

| **miRNA** | **chromosome map** | **Sequence** | **Fold-change** | ***P*-value** | **FDR** |
| --- | --- | --- | --- | --- | --- |
| **Up regulated** | | | | | |
| miR-720 | 3q26.1 | UCUCGCUGGGGCCUCCA | 12.02 | 1.11E-03 | 2.73E-03 |
| let-7a-5p | 9q22.32/11q24.1/22q13.31 | UGAGGUAGUAGGUUGUAUAGUU | 8.86 | 1.24E-03 | 2.73E-03 |
| let-7b-5p | 22q13.31 | UGAGGUAGUAGGUUGUGUGGUU | 7.44 | 4.22E-04 | 2.05E-03 |
| let-7c | 21q21.1 | UGAGGUAGUAGGUUGUAUGGUU | 6.56 | 4.55E-05 | 4.16E-04 |
| miR-26a-5p | 3p22.2/12q14.1 | UUCAAGUAAUCCAGGAUAGGCU | 6.03 | 9.06E-04 | 2.72E-03 |
| miR-23b-3p | 9q22.32 | AUCACAUUGCCAGGGAUUACC | 5.48 | 6.59E-04 | 2.31E-03 |
| miR-23a-3p | 19p13.13 | AUCACAUUGCCAGGGAUUUCC | 5.29 | 8.54E-03 | 1.28E-02 |
| let-7d-5p | 9q22.32 | AGAGGUAGUAGGUUGCAUAGUU | 5.27 | 2.13E-03 | 4.06E-03 |
| miR-145-5p | 5q32 | GUCCAGUUUUCCCAGGAAUCCCU | 5.03 | 1.92E-03 | 3.78E-03 |
| let-7e-5p | 19q13.41 | UGAGGUAGGAGGUUGUAUAGUU | 4.49 | 1.18E-03 | 2.73E-03 |
| miR-103a-3p | 5q34/20p13 | AGCAGCAUUGUACAGGGCUAUGA | 3.07 | 2.50E-05 | 3.15E-04 |
| miR-191-5p | 3p21.31 | CAACGGAAUCCCAAAAGCAGCUG | 3.01 | 1.27E-03 | 2.73E-03 |
| miR-125b-5p | 11q24.1/21q21.1 | UCCCUGAGACCCUAACUUGUGA | 2.91 | 1.12E-02 | 1.56E-02 |
| let-7i-5p | 12q14.1 | UGAGGUAGUAGUUUGUGCUGUU | 2.69 | 8.30E-03 | 1.27E-02 |
| miR-24-3p | 9q22.32/19p13.13 | UGGCUCAGUUCAGCAGGAACAG | 2.60 | 2.44E-02 | 3.01E-02 |
| miR-181a-5p | 9q33.3/1q32.1 | AACAUUCAACGCUGUCGGUGAGU | 2.33 | 2.84E-03 | 5.11E-03 |
| miR-107 | 10q23.31 | AGCAGCAUUGUACAGGGCUAUCA | 2.31 | 2.12E-04 | 1.29E-03 |
| miR-151a-5p | 8q24.3 | UCGAGGAGCUCACAGUCUAGU | 2.15 | 5.99E-04 | 2.22E-03 |
| miR-222-3p | Xp11.3 | AGCUACAUCUGGCUACUGGGU | 2.09 | 2.80E-02 | 3.33E-02 |
| miR-16-5p | 13q14.2/3q25.33 | UAGCAGCACGUAAAUAUUGGCG | 2.05 | 8.52E-04 | 2.68E-03 |
| **Down regulated** | | | | | |
| miR-3141 | 5q33.2 | GAGGGCGGGUGGAGGAGGA | 0.18 | 9.18E-05 | 7.23E-04 |
| miR-1909-3p | 19p13.3 | CGCAGGGGCCGGGUGCUCACCG | 0.17 | 4.62E-05 | 4.16E-04 |
| miR-149-3p | 2q37.3 | AGGGAGGGACGGGGGCUGUGC | 0.17 | 1.07E-03 | 2.73E-03 |
| miR-663a | 20p11.1 | AGGCGGGGCGCCGCGGGACCGC | 0.17 | 1.30E-03 | 2.73E-03 |
| miR-4281 | 5q35.2 | GGGUCCCGGGGAGGGGGG | 0.15 | 1.55E-05 | 3.15E-04 |
| miR-1268a | 15q11.2 | CGGGCGUGGUGGUGGGGG | 0.15 | 1.06E-03 | 2.73E-03 |
| miR-2861 | 9q34.11 | GGGGCCUGGCGGUGGGCGG | 0.15 | 9.53E-03 | 1.40E-02 |
| miR-3185 | 17q21.32 | AGAAGAAGGCGGUCGGUCUGCGG | 0.13 | 1.78E-04 | 1.24E-03 |
| miR-1469 | 15q26.2 | CUCGGCGCGGGGCGCGGGCUCC | 0.12 | 1.83E-03 | 3.72E-03 |
| miR-1908 | 11q12.2 | CGGCGGGGACGGCGAUUGGUC | 0.11 | 5.00E-04 | 2.22E-03 |
| miR-762 | 16p11.2 | GGGGCUGGGGCCGGGGCCGAGC | 0.11 | 1.28E-03 | 2.73E-03 |
| miR-1228-5p | 12q13.3 | GUGGGCGGGGGCAGGUGUGUG | 0.09 | 2.25E-04 | 1.29E-03 |
| miR-3178 | 16p13.3 | GGGGCGCGGCCGGAUCG | 0.07 | 1.19E-02 | 1.63E-02 |
